# Superficial Femoral Artery Pseudoaneurysm and Arterial Wall Destruction After Drug-Coated Balloon Treatment

**DOI:** 10.7759/cureus.10527

**Published:** 2020-09-18

**Authors:** Valentin Golouh, Nina Kobilica, Silva Breznik

**Affiliations:** 1 Department of Radiology, University Medical Centre Maribor, Maribor, SVN; 2 Department of Vascular Surgery, University Medical Centre Maribor, Maribor, SVN

**Keywords:** superficial femoral artery, pseudoaneurysm, drug-coated balloon, paclitaxel

## Abstract

Drug-coated balloon angioplasty may present an efficient alternative to traditional balloon angioplasty and stenting, which suffer from high rates of restenosis and increased risk of stent fractures in the anatomically unfavorable regions, such as the superficial femoral artery in the adductor canal. Although pseudoaneurysms are the most common vascular access site complications, they are considerably rarer at the site of the endovascular treatment. They can be caused by several mechanisms, including stent fractures, usage of oversized balloons, high-pressure inflations, and infections. In addition, paclitaxel, the drug released from drug-coated balloons, may also play a significant role in the formation and exacerbation of pseudoaneurysms. The exact pathophysiology remains unclear, but it may be due to a combination of paclitaxel's suppression of neointimal healing and immune response, cytotoxic properties, and hypersensitivity-related inflammation.

## Introduction

Lower extremity artery disease is estimated to affect approximately 202 million people worldwide, almost one-fifth of them in Europe [1]. Steno-occlusions are often multi-level, with complex calcified morphology [2]. They are frequently located in the superficial femoral artery (SFA) in the adductor canal or the popliteal artery, where the anatomy of the region is unfavorable, as the vessels are exposed to constant dynamic forces [3-4].

The 2017 European Society of Cardiology guidelines recommend the endovascular-first approach only in lesions shorter than 25 cm and the use of drug-coated balloon (DCB) as a consideration in some cases. In longer lesions, bypass surgery is the method of choice. The endovascular approach remains an alternative, as it is more challenging to perform, requiring by-pass landing zones to be protected and stenting avoided [1]. Furthermore, traditional balloon angioplasty, where no stenting is performed after balloon dilatation of the occluded vessel, has up to 40%-60% restenosis, reocclusion, or symptom recurrence rates in complex lesions [5]. In cases of insufficient recanalization, bailout stenting must be performed. Due to the suppression of intensive neointimal hyperplasia occurring after angioplasty, DCBs present a potentially cost-effective way to reduce the need for stenting and improve patency [3,6-7]. However, concerns of potential paclitaxel toxicity with paclitaxel-coated balloons emerged recently [2]. We present a case of a pseudoaneurysm after DCB angioplasty of an occluded SFA in the adductor canal.

## Case presentation

**Figure 1:**
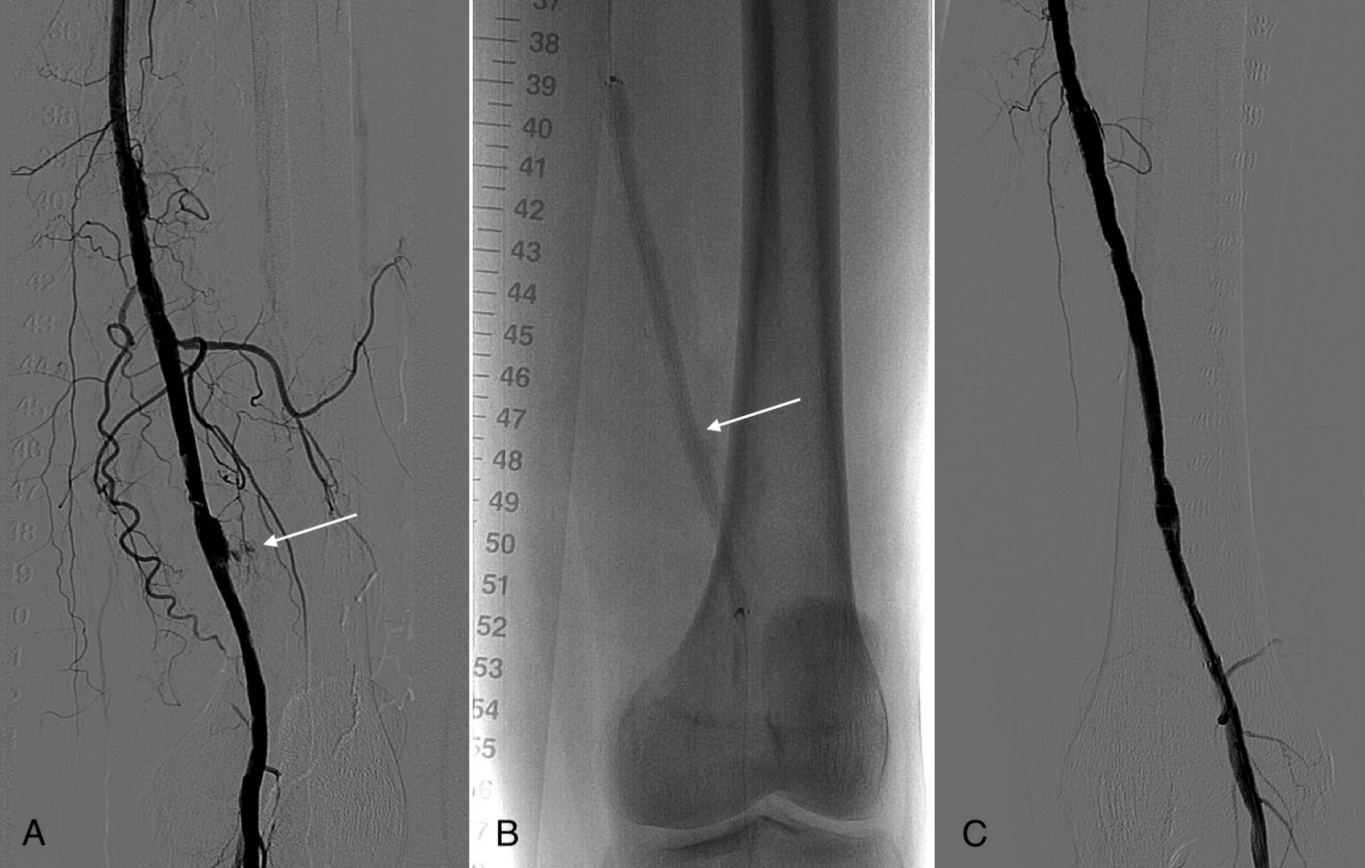
(A) Control angiography during PTA revealed contrast extravasation (arrow) in the area of the SFA after the recanalization and dilatation with a 5 mm balloon catheter and a 6 x 150 mm DCB. (B) A 6 x 150 mm Viabahn stent-graft (arrow) at the place of the ruptured SFA wall can be seen on the control angiography. (C) No contrast leakage can be seen on the control angiography after PTA and stenting. PTA - Percutaneous transluminal angioplasty; SFA - Superficial femoral artery; DCB - Drug-coated balloon

A 72-year old woman with claudication underwent endovascular treatment. The left SFA was first dilatated with a 5 mm balloon catheter, and an additional dilatation with a 6 x 150 mm DCB was performed. Extravasate in the vicinity of the SFA was seen on the control angiography (Figure 1A); therefore, a 6 x 150 mm Viabahn stent-graft (Gore Medical, Flagstaff, Arizona) was inserted (Figure 1B). No leakage was observed on the angiography, and the patient was discharged the next day (Figure 1C).

**Figure 2:**
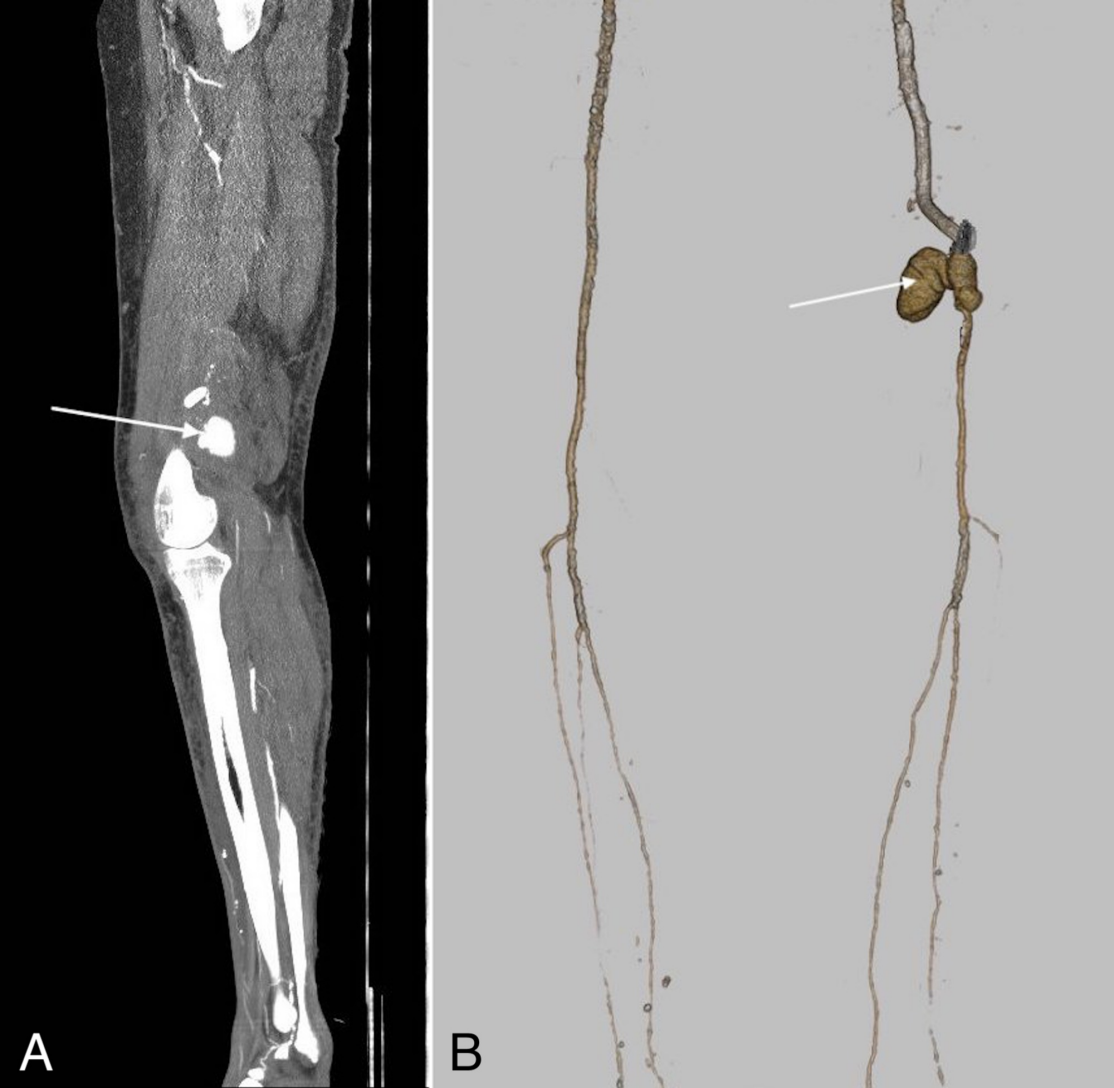
(A) CTA of the left leg shows a pseudoaneurysm (arrow) in the popliteal fossa. (B) A 3,5 x 3,5 cm pseudoaneurysm (arrow) behind the caudal end of the stent-graft placed in the SFA can be observed on the reconstruction. SFA - Superficial femoral artery, CTA - Computed tomography angiography

**Figure 3:**
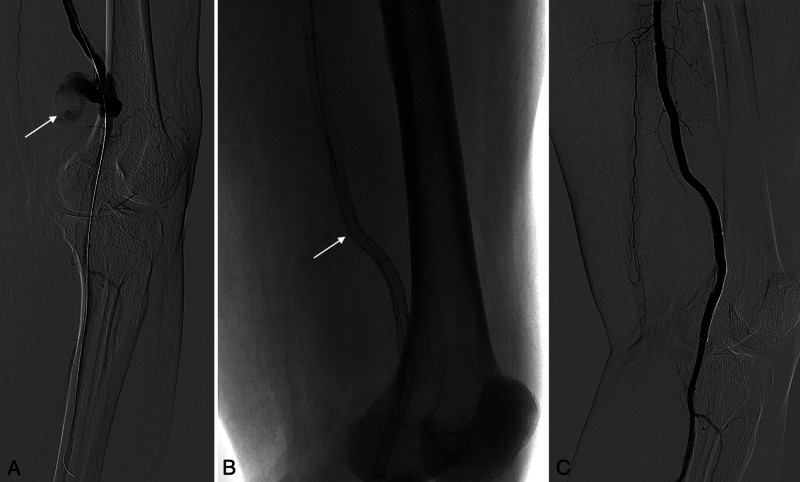
(A) Angiography of a pseudoaneurysm (arrow) before stenting. (B) Another 6 x 100 mm Viabahn stent-graft was placed caudally to the pseudoaneurysm and additionally reinforced with a 5,5 x 150 mm Supera stent (arrow). (C) No contrast leakage is observed on the control angiography.

Two months later, the patient returned with edema of the left leg, which was painful upon palpation and spanned from the medial side of the knee towards the shin. A retraction of the stent-graft and a pseudoaneurysm were seen on computed tomography angiography (CTA) (Figures 2A-2B). A guidewire passage was performed through the inserted Viabahn stent-graft and the pseudoaneurysm into the popliteal artery. Another Viabahn stent-graft was then inserted. Lastly, both Viabahn stent-grafts were overstented with a Supera stent (Abbott Vascular, Abbott Park, Illinois, to gain more radial force (Figures 3A-3C).

**Figure 4:**
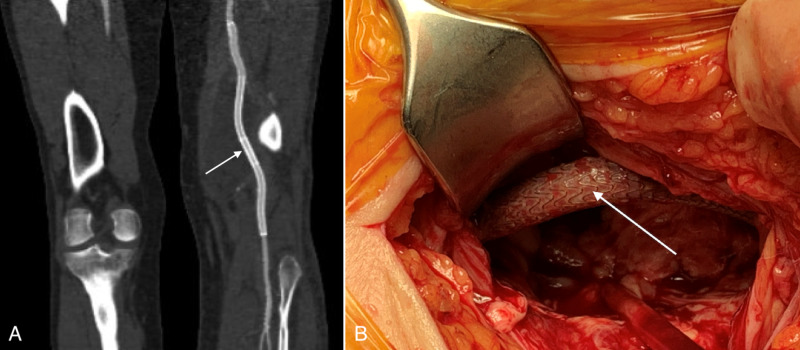
(A) Tortuous stent-grafts and the stent (arrow) can be seen on the control CTA. (B) Upon surgical revision, a complete destruction of the arterial wall and a free-standing stent-graft (arrow) were observed in the adductor canal. CTA - Computed tomography angiography

CTA after four months showed a deviated, tortuous distal SFA and the first segment of the popliteal artery, where stent-grafts had been placed (Figure 4A). An infected collection persisted behind the caudal end of the stent-graft. Surgical exploration revealed an absence of the arterial wall and a free-standing stent-graft in the adductor canal (Figure 4B). However, blood flow to the foot was sufficient, and intraoperative contrast-enhanced ultrasound showed no contrast leakage.

**Figure 5:**
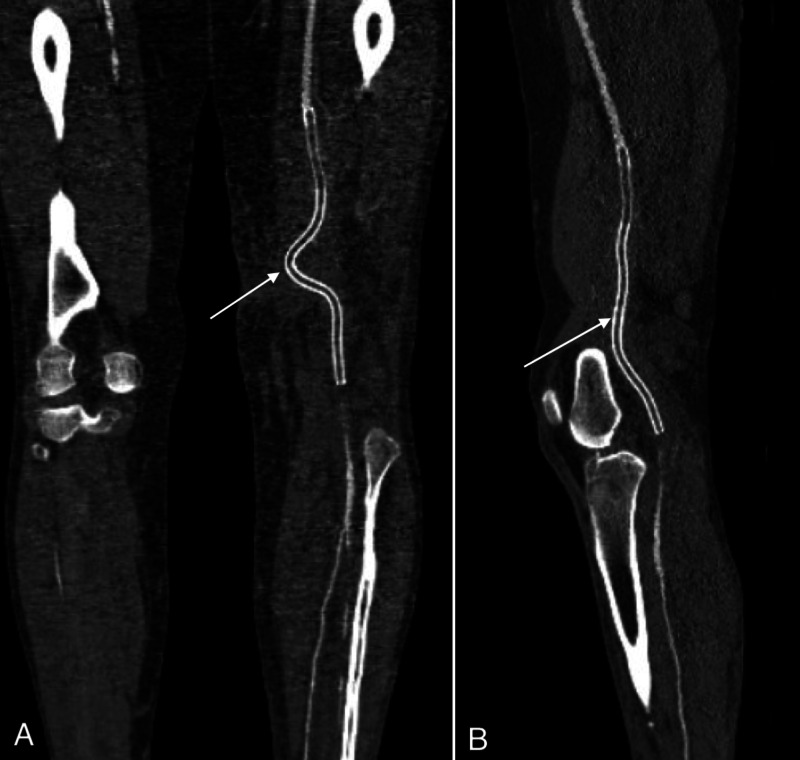
(A, B) Control CTA shows occluded and cranially retracted stent and stent-grafts (arrow). CTA - Computed tomography angiography

**Figure 6:**
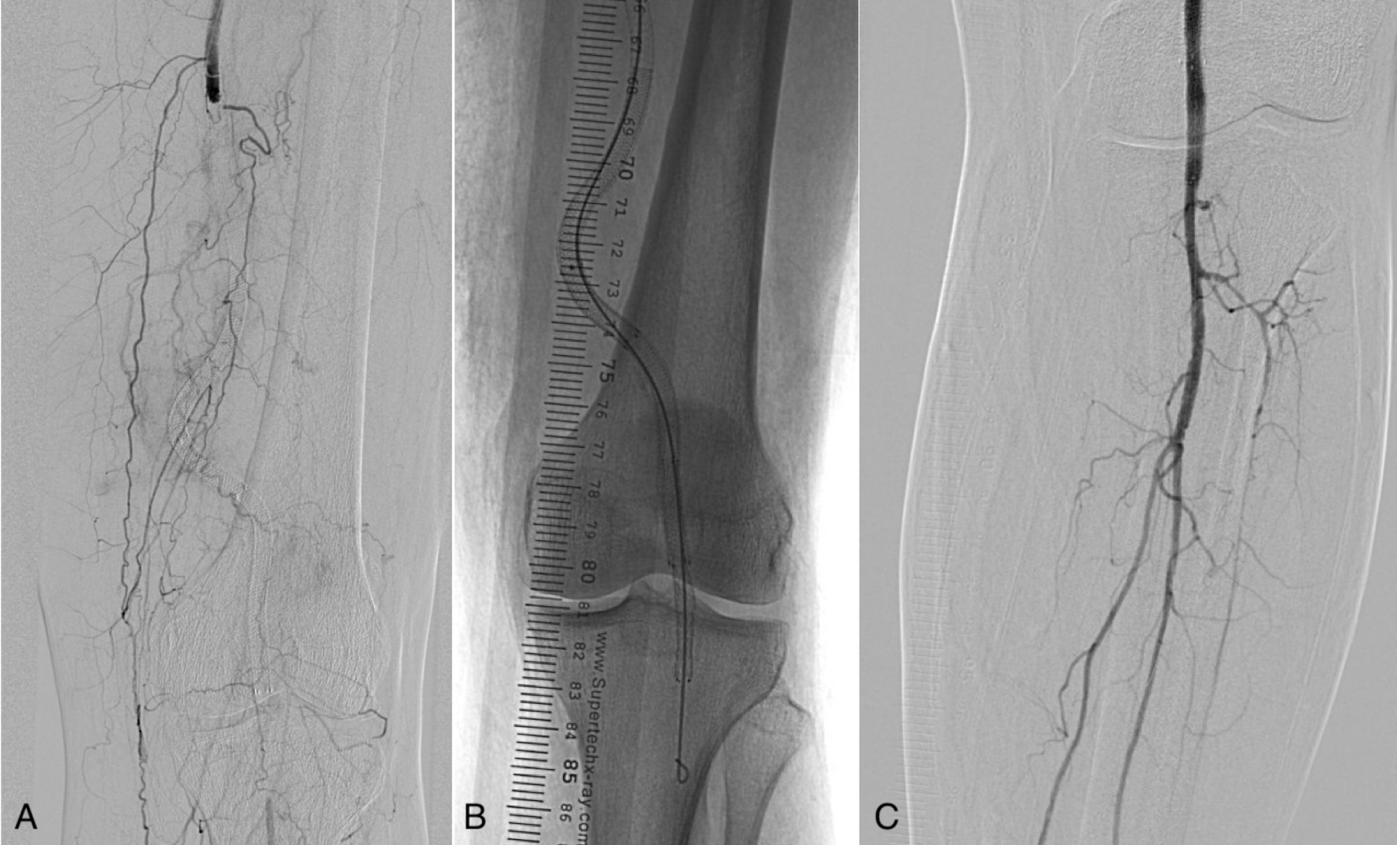
(A) Angiography shows occluded and cranially retracted stent and stent-grafts (B) After thrombectomy and aspiration of the distal emboli, in-stent restenting with a 6 x 80 mm stent was performed on the caudal end of the previously inserted stent-grafts. (C) Control angiography after the successful recanalization of the occlusion.

The patient returned six months later with oedematous, painful toes and severe walking difficulties. Previously purulent discharge from her postoperative left thigh fistula was now bloody. CTA showed no pseudoaneurysm but both stent-grafts and the stent occluded and cranially retracted (Figures 5A-5B). Endovascular recanalization and in-stent restenting were performed (Figures 6A-6C).

**Figure 7:**
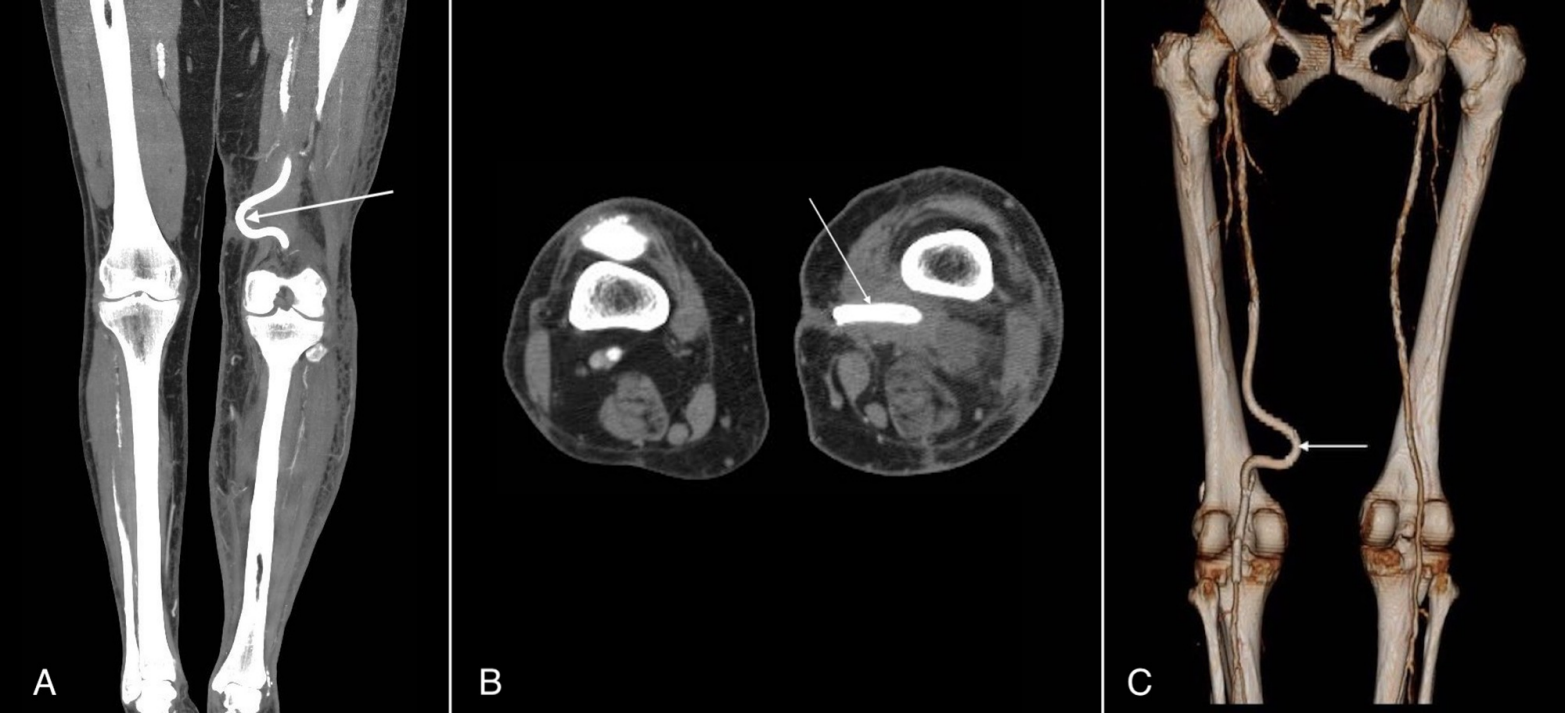
(A, B, C) CT one year after the initial perforation and stenting shows severely deviated and tortuous stent-graft (arrows) in the left adductor canal, bulging into subcutaneous layers merely 7 mm from the skin’s surface; without any pseudoaneurysm. CT - Computed tomography

One month later, the discharge from the hip fistula was again bloody and the patient complained of severe pain in her left foot that prevented her from sleeping at night. CTA revealed severely tortuous stent-grafts, bulging into subcutaneous layers, 7 mm from the skin’s surface, with a large surrounding hematoma (Figures 7A-7C). Prompt surgical explantation and auto-venous femoropopliteal bypass were performed.

## Discussion

Pseudoaneurysms occur after a disruption of the arterial wall and blood leakage. Pressure from the resulting hematoma causes inflammation of the surrounding tissue and a pseudocapsule connected to the artery forms from soft tissue or adventitia. Pseudoaneurysms may occur on any vessel and due to a myriad of conditions, including dissection, inflammatory diseases, pregnancy, and trauma. They are the most common vascular access complication, with an incidence of 0.5% to 2% [8-10]. The risk increases with sheath size, age, body mass index (BMI), female gender, concurrent anticoagulation, arterial calcification, combined arterial and venous puncture, and cannulation of arteries other than the common femoral artery [11].

Patients often remain asymptomatic but may present with pain and swelling due to hematoma. Doppler sonography is the preferred diagnostic method. Additionally, CTA may be needed for confirmation and therapy planning but should be employed warily, as it carries radiation exposure, contrast nephrotoxicity, and higher cost. Treatment options include ultrasound-guided thrombin injection (UGTI), ultrasound-guided compression, open surgical repair, and endovascular therapy. Though UGTI is the recommended method, it carries a risk of thromboembolic events and should be considered with care in cases of severe peripheral arterial disease. Growing pseudoaneurysms can rupture and result in life-threatening bleeding. Other complications include infection, distal embolization, and compression of surrounding vessels [9].

Only a few SFA pseudoaneurysms have been described, none after a DCB angioplasty [8,12]. Cardiologic literature has described comparably more pseudoaneurysms, several after drug-eluting stent implantation [10,13-15]. The causes of vessel injury included oversized balloons, high-pressure inflation, stent fracture, or infection [13]. Although the precise pathophysiology is unknown, paclitaxel may be implicated due to a combination of cytotoxic effects on the vascular wall, hypersensitivity-related extensive inflammation, and delayed neointimal healing [6,16-17].

It may also represent the mechanism of complete SFA arterial wall destruction after rupture during DCB angioplasty and stent-grafting in our case. Paclitaxel-related immunosuppression could have additionally promoted pseudoaneurysm infection and exacerbated the inflammation [13]. Lastly, the dynamic mechanical strain on a weakened arterial wall may also have played a role, as structures in the adductor canal are exposed to constant compression, distention, flexion, and torsion [3-4].

## Conclusions

In the setting of pain, hematoma, or swelling after a percutaneous intervention, a pseudoaneurysm should be suspected. The role of DCBs and the potential toxicity of paclitaxel remain to be explored by future research. However, caution and frequent monitoring after DCB usage and the stenting of femoropopliteal arteries may be appropriate to avoid potential adverse outcomes in these patients.
